# Renaming Medullary Cystic Kidney Disease: A Review of Semantic Nomenclature

**DOI:** 10.7759/cureus.96561

**Published:** 2025-11-11

**Authors:** Kevin Kuang, Sonika Vatsa, Rahul Ramakrishnan, Ahmik Shenoy, Mohammadali M Shoja, Ghaith Al-Eyd

**Affiliations:** 1 Medical Education, Nova Southeastern University, Dr. Kiran C. Patel College of Allopathic Medicine, Fort Lauderdale, USA; 2 Internal Medicine, Rowan University, School of Osteopathic Medicine, Stratford, USA; 3 Medical Education, Midwestern University, Arizona College of Osteopathic Medicine, Glendale, USA

**Keywords:** autosomal dominant tubulointerstitial kidney disease, kidney cysts, mckd, medullary cystic kidney disease, nephronophthisis

## Abstract

Medullary cystic kidney disease (MCKD) was originally described and designated with this nomenclature based on the etiology and the related clinicopathological features recognized during the mid-20th century. The designation of the MCKD term has stemmed from a set of morphological features characterized by the presence of epithelial-lined cysts ranging in size from a few microns to 1 cm in diameter, primarily located at the corticomedullary junction of the kidney. However, this term does not adequately represent the expanding knowledge about the kidney condition it describes, including its genetically based etiopathogenesis and the related clinicopathological correlations. The need for a more patient-centered and specific terminology has been recognized by the medical community and has necessitated reviewing the MCKD nomenclature. The proposed renaming of the condition to autosomal dominant tubulointerstitial kidney disease aligns closely with the genetic and pathological foundations of the disease. This renaming aims not only to enhance awareness about this kidney condition but also to improve its diagnostic accuracy and treatment strategies. This article highlights the historical observations related to MCKD and underscores the importance of adopting a revised and precise terminology.

## Introduction and background

**Figure 1:**
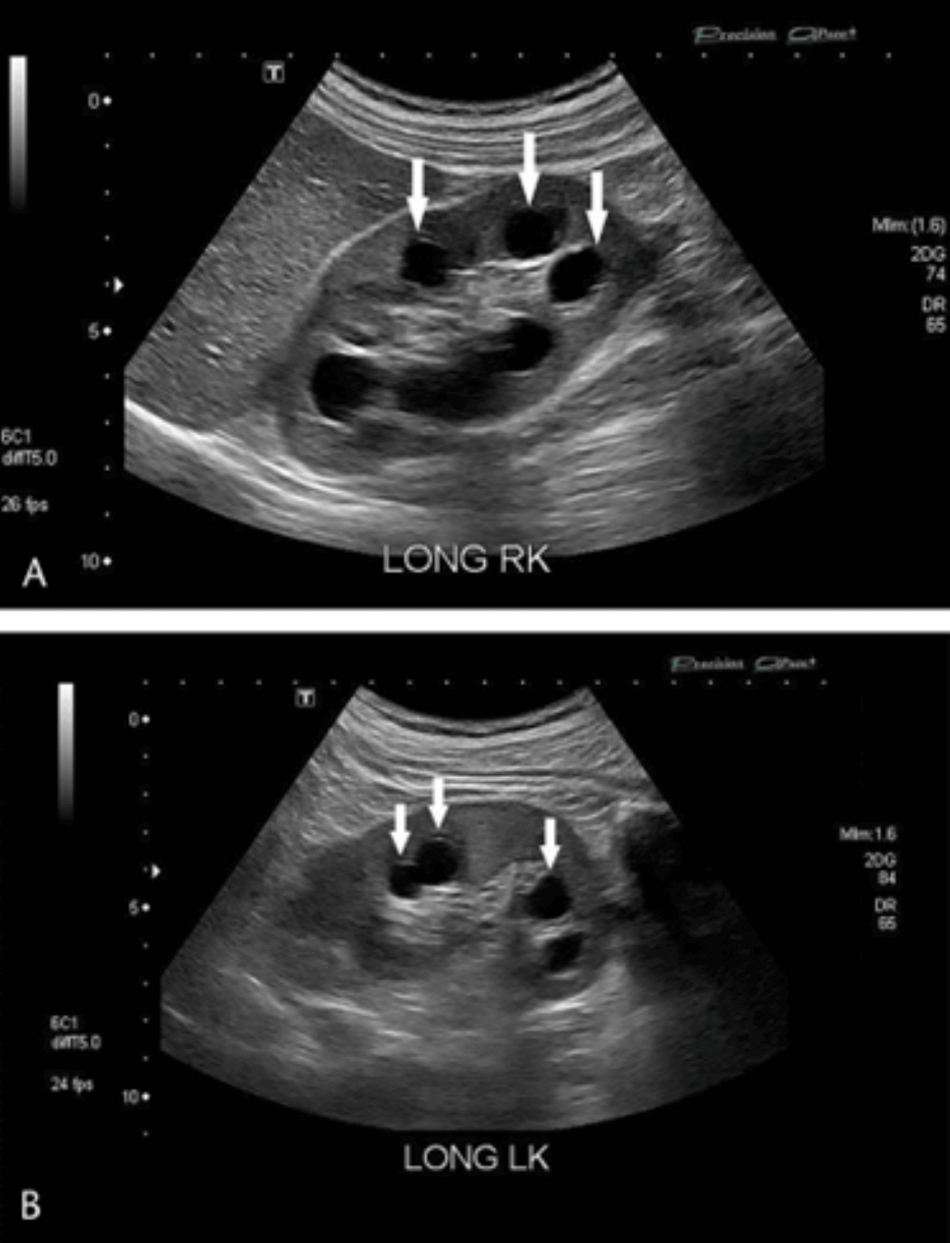
Longitudinal kidney ultrasound of a patient with medullary cystic kidney disease (MCKD). This image depicts multiple medullary cysts in the right (A) and left kidney (B). (Reproduced from Whang et al., with permission from Wolters Kluwer Health, Inc.) [2].

Medullary cystic kidney disease (MCKD) is a kidney pathology that is often paired with nephronophthisis (NPH), given their similar histological and clinical characteristics. Both conditions manifest as bilateral small cysts in the kidney medullary and corticomedullary regions (Figure 1) [1,2]. The kidney lesion associated with these conditions eventually evolves to tubulointerstitial sclerosis that culminates in kidney failure.

Although both MCKD and NPH share some clinicopathological features, ongoing studies have been showing some distinct differences in their clinical presentations and age of onset, besides their different mode of inheritance (autosomal dominant MCKD versus autosomal recessive NPH). The goal of this paper is to trace the historical evolution, clinical characterization, and molecular understanding of MCKD, and to examine its longstanding conflation with NPH. By exploring the shared and divergent clinical, histopathological, and genetic features of these two pathologies, the paper highlights how the initial terminology of MCKD has become increasingly obsolete. In light of recent advances in the pathophysiological understanding of the disease and the growing emphasis on patient-centered, precise medical nomenclature, the paper supports the transition toward the term autosomal dominant tubulointerstitial kidney disease (ADTKD). It further explores the broader implications of this terminological shift on diagnosis, patient care, research, and healthcare policy, advocating for standardized nomenclature to improve diagnostic clarity and reduce misclassification across clinical and research settings.

## Review

Discovery of MCKD and origin of the term

The origin of MCKD is traced back to the middle of the 20th century, when the first cases of medullary cysts of the kidney were documented. The first report dates back to 1945 when Graham and Smith described a child presenting with refractory anemia, azotemia, and congenital medullary kidney lesions [3]. In particular, Graham and Smith discovered multiple cysts originating in the medulla, mainly in the medullary pyramids, without any kidney cortex involvement. The impact of the medullary cysts was significant as the expanding cysts caused swelling in the kidneys due to increased intratubular pressure. At the time of that case, the authors were unable to conclude how multiple cysts arose in a young patient and whether the severe anemia had any correlation with the medullary cysts.

A few years later, in 1951, Fanconi and his colleagues described an autosomal recessive kidney disease with similar manifestations that occurred prior to puberty in two different families [4]. Those affected children presented with kidney failure, reduced kidney size, anemia, and an inability to concentrate urine appropriately. It was noted that kidney failure was not associated with hypertension, proteinuria, or hematuria. The term familial juvenile NPH (FJN) was applied to describe that condition. While Fanconi et al. did not explicitly describe cysts in their study, the photograph of the kidney from one of their cases revealed the presence of several cysts [5]. In 1962, Sydow and Ranström examined eight other families affected by autosomal recessive FJN and noted consanguinity in two cases among the parents of affected children [6]. They also suggested that environmental factors might have contributed to the initiation and progression of the condition.

In 1952, Murphy and his colleagues reported a patient with salt-losing nephritis presenting with paresthesia, hematemesis, bleeding gums, and cramping [7]. When that patient had eventually passed away due to a seizure, an autopsy was performed, and it was noted that there were multiple subcortical and medullary cysts in the kidney filled with a watery and colorless liquid. The cysts in that lesion did not impede the collecting tubules, but they did impact the papillae. It was subsequently concluded that the patient had medullary cysts in the context of chronic pyelonephritis.

**Figure 2:**
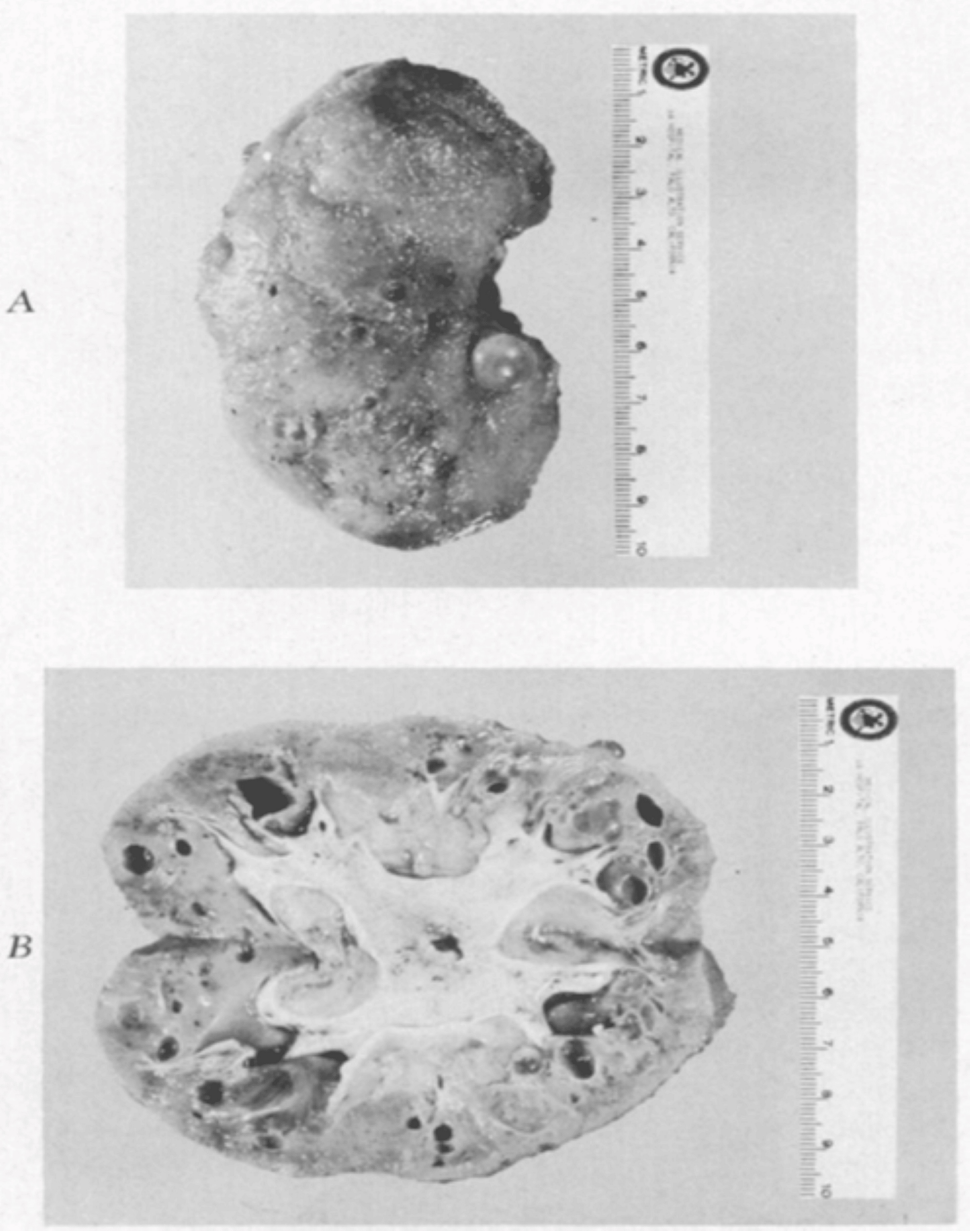
Gross pathology of medullary cystic kidney disease (MCKD). Panel A displays the entire right kidney, and Panel B shows a cut section. (Reproduced from Goldman et al. with permission from the Publishing Division of the Massachusetts Medical Society) [9].

The definitive discovery of MCKD occurred in 1962, when Strauss examined a cohort of 18 young adults (median age of 27 years) who exhibited medullary cysts and kidney failure [8]. Through comprehensive analysis of the clinical features and the underlying pathological changes, Strauss coined the term "medullary cystic disease" to describe that condition. The clinical features encompassed a gradual presentation of azotemia, anemia, and salt-wasting, with no unusual sediment or albumin in the urine. Strauss was unable to unravel the pathogenesis of this condition or its inheritance pattern. Strauss's microscopic analysis of kidney tissue closely mirrored the findings reported by Goldman [9]. The analysis revealed a variety of cysts, ranging from a few microns to 1 cm in diameter, predominantly situated at the corticomedullary junction (Figure 2) [8,9]. The cysts were lined by a single layer of cuboidal or flattened epithelial cells.

In the years following Strauss' paper, the genetic inheritance of MCKD remained elusive due to the scarcity of documented patients with the condition. In 1966, Goldman and his team undertook an investigation into the inheritance pattern of MCKD. Their study involved an examination of a male patient with MCKD and five successive generations of his family, encompassing a total of 60 individuals [9]. After reviewing Strauss' data and analysis of the findings from the index family, they came to the conclusion that the genetic inheritance of MCKD follows an autosomal dominant pattern. They also noted that an autosomal recessive mode of inheritance may have occurred in isolated instances not addressed by their study. Examination of kidney tissue samples revealed consistent features such as lymphocytic and plasma cell infiltrates, hyalinized glomeruli, and medullary cysts. Goldman theorized that MCKD might be linked to infection-induced cysts and medullary fibrosis.

In the meantime, the prevailing theory posited that MCKD, stemming from a multitude of etiologies such as genetic or nongenetic factors, acquired toxins, or infections, predominantly affects the medullary region of the kidneys, with cortical changes emerging as secondary processes [10]. In 1968, Handa and Tennant reported two siblings with MCKD who exhibited kidney findings similar to those described by Strauss [11]. Both siblings had kidneys with cortical scarring and medullary cysts. Handa and Tennant reviewed existing theories on the onset and progression of MCKD. Among the proposed theories, inborn errors of metabolism were linked to the accumulation of nephrotoxins, and the individual’s primary or vestigial tubules, normally destined for cystic degeneration, may have persisted instead, resulting in early-onset cystic degeneration. Since those early studies and through subsequent years, research has continued to advance the understanding of the pathologic basis of MCKD through morphologic and genetic analyses. 

Medullary cystic kidney disease versus nephronophthisis

The similarity of the manifestations of both MCKD and FJN has led to describing these two conditions as having a virtually identical clinical presentation [10]. To address this debate, Strauss and Sommers, in 1967, compared the clinicopathological features of six patients diagnosed with FJN in Norway and Sweden (referred to as the "European cases") and three patients diagnosed with medullary cystic disease in the United States (referred to as the "American cases") [5]. Based on the limited number of cases examined, Strauss and Sommers noted that both conditions were indistinguishable clinically as well as at the level of light microscopic morphology. The available evidence was insufficient to definitively determine the genetic transmission pattern of the two diseases. Considering the potential clinicopathological identity of the two diseases, Strauss and Sommers proposed that the term "medullary cysts of the kidney" should be retained instead of "familial juvenile nephronophthisis." They argued that the latter designation was "misleading," "unduly restrictive," and merely a "high-sounding tongue-twister of dubious connotation.”

By the 1970s, it had been well established that the recessive NPH and dominant MCKD share common kidney histopathological features, characterized by a triad of tubular basement membrane disintegration, tubular atrophy accompanied by cyst formation, and interstitial cell infiltration leading to fibrosis [12]. With the increasing evidence and reported series, it became apparent that the two conditions could be distinguished based on the mode of inheritance and age of onset [13]. In 1977, Chamberlin et al. advocated that FJN and MCKD “very likely” represented a single disease entity, occurring as a juvenile-onset, autosomal recessive form (i.e., FJN) and an adult-onset, autosomal dominant form (i.e., MCKD) [14]. The terms NPH and MCKD were used interchangeably by many authors. Specifically, FJN was denoted as juvenile-onset, autosomal recessive MCKD, whereas autosomal dominant MCKD was characterized by the presence of cysts occurring later in life at the corticomedullary boundary area [15]. Due to similarities in clinical presentation and histopathology, in 1987, the Section on Urology of the Committee on Terminology, Nomenclature, and Classification of the American Academy of Pediatrics recommended the term “'juvenile nephronophthisis-medullary cystic disease complex” [16,17]. As more was discovered, these two pathologies were found to be distinct in their inheritance, presentation, and age of onset.

Molecular studies and disease characterization

**Figure 3:**
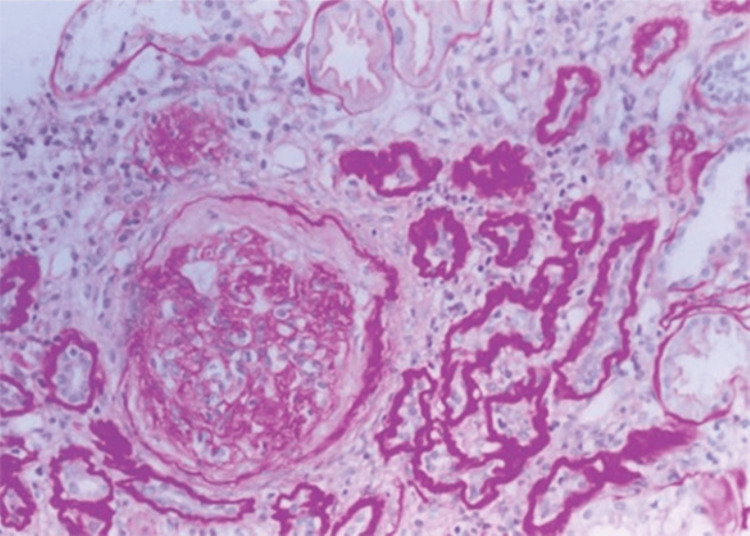
Kidney biopsy histopathology changes in autosomal dominant medullary cystic kidney disease (MCKD). This image demonstrates characteristics of tubular atrophy, lymphocytic infiltration, and interstitial fibrosis. (Reproduced from Stavrou et al., International Society of Nephrology, with permission from Elsevier) [20].

The 1990s marked a significant milestone in unraveling the intricate FJN/MCKD complex, as significant strides in molecular genetics culminated in the precise mapping of the FJN gene to chromosome 2p and the identification of the gene associated with dominant MCKD on chromosome 1 [18,19]. A study of 186 individuals from Cyprus employed molecular analysis to validate the MCKD1 locus (chromosome 1q21) linkage [20]. Examination of kidney tissue under light microscopy revealed lymphocytic infiltration, interstitial fibrosis, and tubular atrophy (Figure 3). The presence of medullary or corticomedullary cysts, detected in 40.3% of patients, was deemed unreliable as a definitive diagnostic marker for MCKD [20]. It was proposed that leveraging genetic tools like DNA linkage analysis or genetic mapping to establish autosomal dominant inheritance could facilitate the accuracy and effectiveness of MCKD diagnosis.

**Figure 4:**
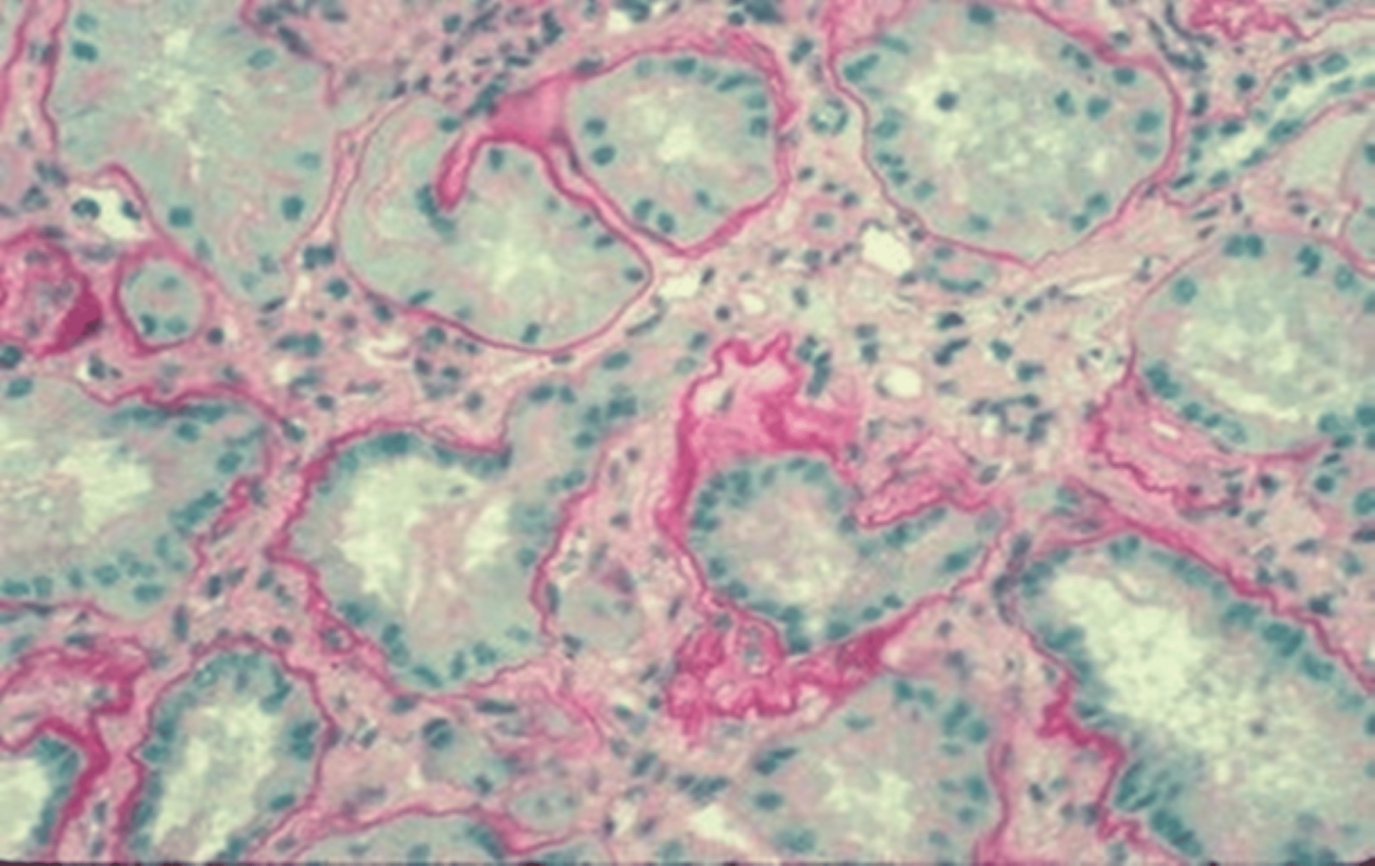
Kidney biopsy histopathology changes in nephronophthisis. This image shows interstitial fibrosis, thickened basement membrane, and tubular alterations. (Reproduced from Salomon et al. under Creative Commons CC BY license) [16].

The autosomal recessive NPH was characterized as having three distinct subtypes: infantile, adolescent, and juvenile. The onset of kidney failure and genetic mutations associated with each subtype vary. The infantile type is linked to mutations in the NPH2 gene, where patients exhibit kidney cysts, severe hypertension, enlarged kidneys, and eventually develop kidney failure before the age of four. The adolescent subtype is associated with a mutation in the NPHP3 gene, leading to kidney failure around the age of 20. Lastly, the juvenile type, which is the most common type of NPH, is associated with nine genetic mutations, resulting in a diverse range of clinical manifestations. Patients with the juvenile subtype develop kidney failure in their second decade of life and may present with small medullary cysts, tubular atrophy, thickened basement membrane, and interstitial fibrosis (Figure 4) [1-2,16].

**Figure 5:**
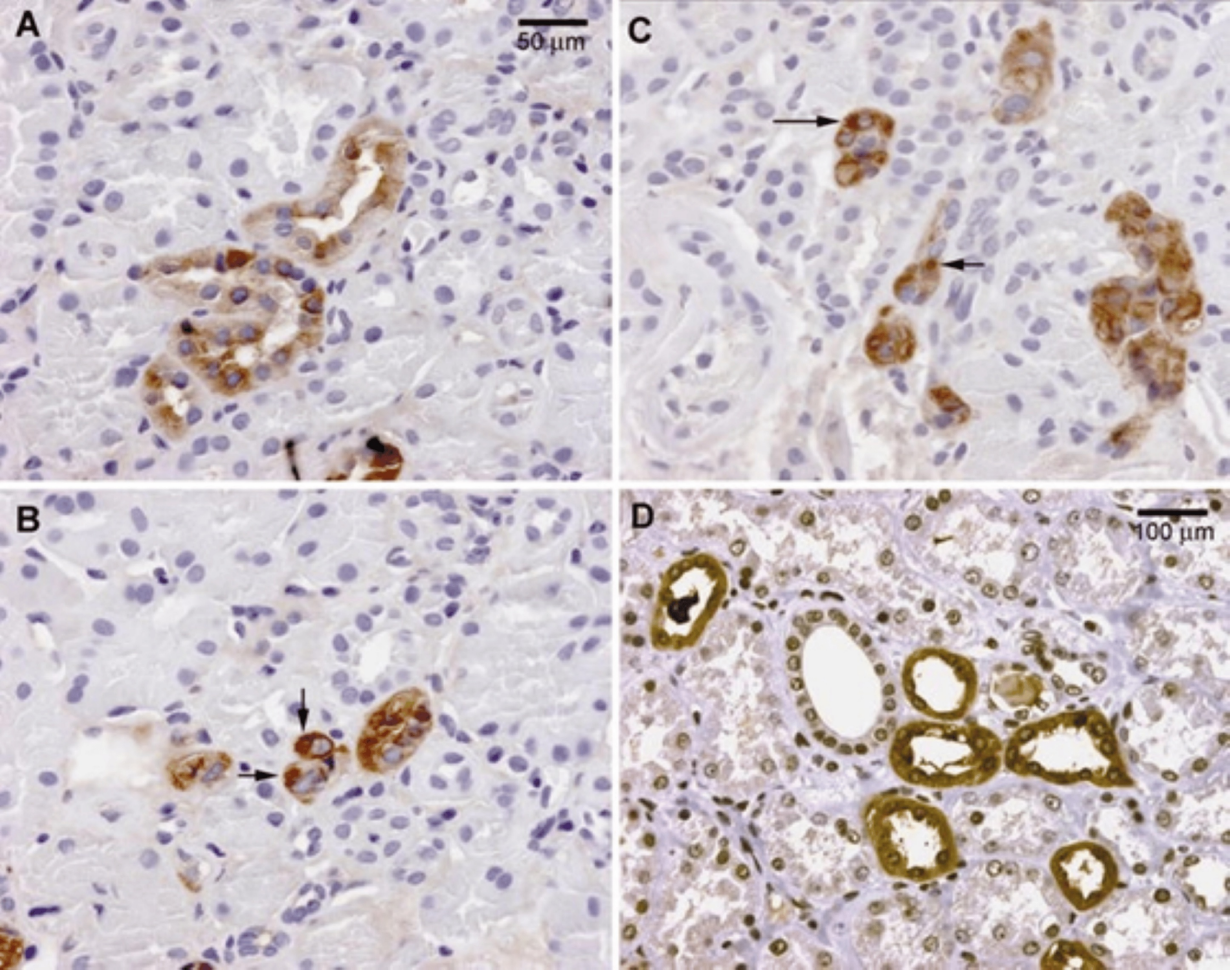
Renal immunohistochemical staining of uromodulin in a patient with type 2 medullary cystic kidney disease (MCKD). Panels A, B, and C depict abnormal staining with dense deposits in the patient, while Panel D shows diffuse staining of uromodulin in a healthy control. (Reproduced from Bleyer et al., International Pediatric Nephrology Association, with permission from Springer Nature) [29].

Compared to NPH, MCKD is an autosomal dominant disorder that results in kidney failure in adulthood without extra-kidney manifestations. Unlike NPH, the presence of medullary cysts is not required for MCKD diagnosis. Traditionally, MCKD has been classified into two forms: MCKD type 1 and type 2, and currently, additional variants have been recognized. Table 1 summarizes the main MCKD-related genetic mutations, their corresponding pathologic mechanisms, and the affected kidney cells [21-27]. Type 1 MCKD is associated with mutations in the MUC1 gene, and it leads to kidney failure at around the age of 60 [21]. Type 2 MCKD is linked to mutations in the UMOD (Tamm-Horsfall) gene and leads to kidney failure at a younger age, around 30 years. Sometimes referred to as uromodulin-associated kidney disease (UAKD), this type also presents with early hyperuricemia clinically mimicking another kidney pathology, familial juvenile hyperuricemic nephropathy [28]. However, the immunohistochemical staining of uromodulin in type 2 MCKD shows dense deposits in tubular cells and irregular staining (Figure 5) [29]. Mutations in the REN gene have also been identified and were also linked to the development of MCKD by reducing the processing of preprorenin [1,16,30]. In addition, recent studies have described the involvement of the HNF1B gene in the pathogenesis of MCKD, which mainly includes tubulointerstitial fibrosis [22]. It appears that mutations in MUC1, UMOD, and REN genes first injure epithelial tubular cells, initiating a cascade of cellular stress and dysfunction. The tubular response eventually leads to a secondary reaction in the interstitium [31-36].

**Table 1:** The genetic mutations involved in medullary cystic kidney disease (MCKD). This table summarizes the MCKD-related genetic mutations, their corresponding pathologic mechanisms, and the affected renal cells [21-27]. HNF1B: hepatocyte nuclear factor 1-beta; MUC1: mucin 1; REN: renin; UMOD: uromodulin

Gene Mutation	Pathogenic Mechanism	Affected Kidney Cells
MUC1 Gene Mutation (Type 1 MCKD/ADTKD)	Abnormal mucin-1 protein accumulates, resulting in cellular toxicity and progressive tubulointerstitial fibrosis. Often it is a cytosine duplication that results in a frameshift mutation	Cytoplasm of distal tubular epithelial cells
UMOD Gene Mutation (Type 2 MCKD/ADTKD)	Missense mutations result in protein misfolding, resulting in protein retention within the endoplasmic reticulum (ER). This ER retention can activate the unfolded protein response, causing chaperone upregulation and ER stress.	Endoplasmic reticulum of thick ascending limb epithelial cells
REN Gene Mutation	Renin production/secretion becomes impaired, leading to tubulointerstitial fibrosis. Interruptions in the signal peptide can disrupt ER translocation, leading to intracellular retention of preprorenin or prorenin.	Juxtaglomerular cells of the juxtaglomerular apparatus
HNF1B Gene Mutation	Disturbed nephrogenesis and maintenance of tubular function caused by abnormal tubular differentiation. Changes include enhanced epithelial-mesenchymal transition leading to fibrosis.	Renal tubular cells across the nephrons and collecting ducts

In a nutshell, although there are differences between NPH and MCKD, these two kidney pathologies do share some overlapping features. Moreover, MCKD is considered a terminological umbrella that encompasses various kidney pathologies sharing comparable manifestations, and thus, it is referred to by several other names. Due to this lack of clarity regarding MCKD as an overarching pathology, the delineation, diagnosis, and treatment of the conditions encompassed by the MCKD term can be challenging.

Push for medullary cystic kidney disease name change

With the recent advances in Pathology and the related growing knowledge about the different entities of kidney disease, a more standardized and precise terminology has been sought to describe the MCKD lesions. In 2019, the “Kidney Disease: Improving Global Outcomes” (KDIGO) initiative began creating a consensus on acceptable nomenclature. The group identified a lack in the American Medical Association Manual of Style (10th edition) specific to kidney pathologies and therefore created the following criteria in their pursuit of universal language: patient-centeredness and terminological precision. With this in mind, KDIGO began overhauling the current disease names and descriptions. For example, when describing kidney failure, the committee agreed to avoid using the term “end-stage” due to its vague and inconsistent usage. Additionally, it seemed to create a stigma among patients that perhaps it was the end for them. More specific verbiage, such as kidney failure and a numerical hierarchical description of the failure, was deemed more appropriate [37].

However, the lack of a suitable name for MCKD was recognized much earlier owing to the magnitude of its consequences. Terminological inadequacy stems from the fact that large or small cysts are not required for diagnosis and, in fact, are relatively uncommon manifestations [36]. Additionally, the kidney interstitium and basement membrane are more commonly affected than the medulla. While the initial study of Graham and Smith in 1945 showed cysts in a pediatric patient, contemporary research has shown that arteriolar thickening and hyalinosis with interstitial fibrosis were more common pathologic findings. These findings resonate with the concept of the original nomenclature of the non-genetic “Tubulointerstitial Disease” also known as “Tubulointerstitial nephritis”. That concept is based on the fact that the disease process primarily involves the interstitium and tubules and relatively spares the glomeruli until late stages [38].

**Figure 6:**
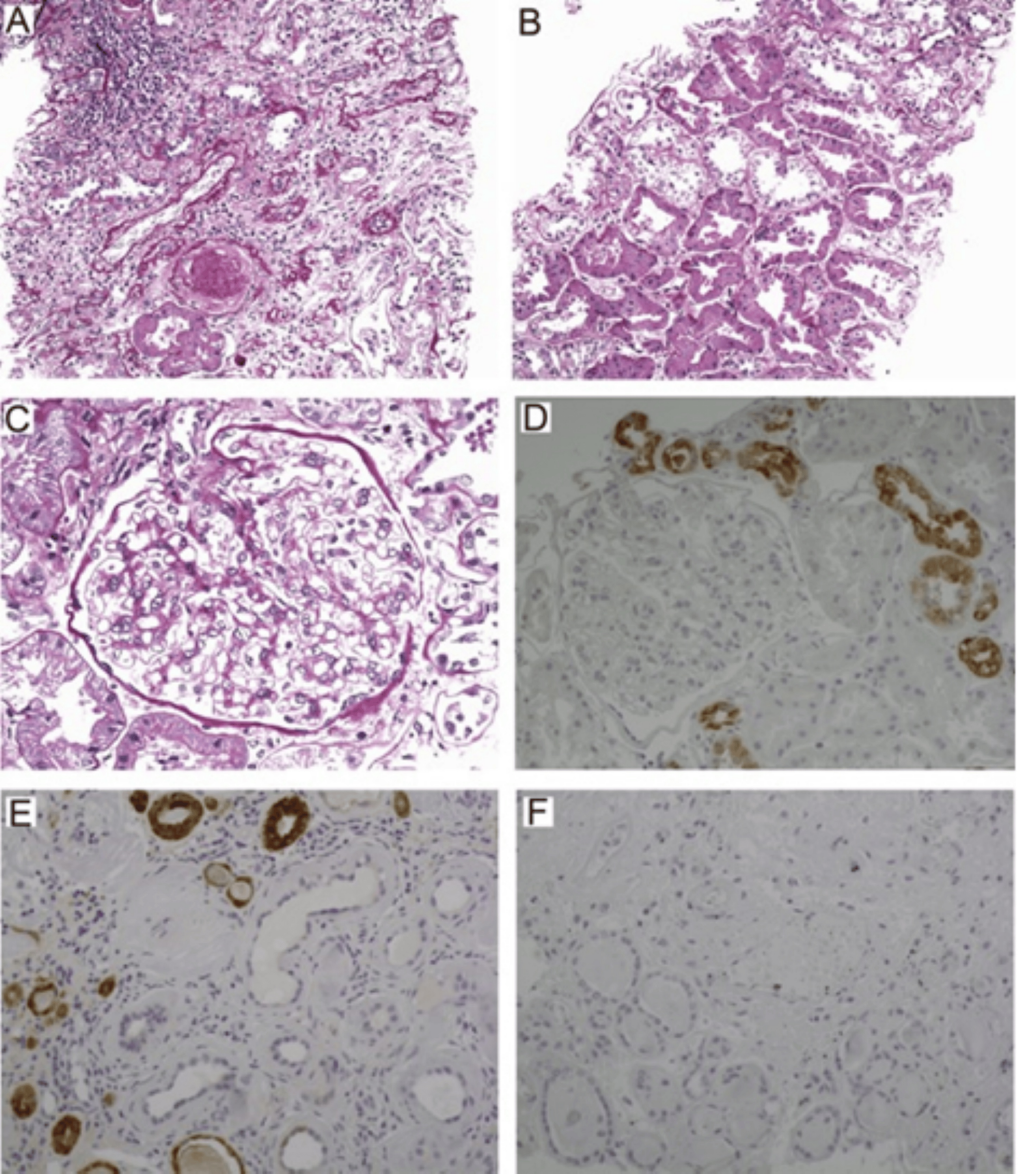
Kidney biopsy histopathology changes in type 1 medullary cystic kidney disease (MCKD). (A) Significant cortical scarring with lymphocytic infiltration; (B) renal cortex with normal tubular structure in PAS staining; (C) normal glomeruli. Utilizing antibody PA4301, (D) demonstrates notable granular staining of MUC-1fs in patient tissue, while (E) exhibits a positive control with similar staining. (F) represents a negative MUC-1fs result for the negative control. (Reproduced from Yu et al., the National Kidney Foundation, Inc., with permission from Elsevier) [39].

The failure to consider the genetic basis of the disease was another issue with the MCKD nomenclature. Among the four key genes (MUC1, UMOD, REN, and HNF1B) involved in the pathogenesis of MCKD, MUC1 and UMOD lead to the predominant forms. The MUC1 mutation is traditionally denoted as mucin 1 kidney disease or MCKD type 1 (Figure 6) [39]. Hence, due to this genetic heterogeneity and subsequent variability in clinical manifestations, the term MCKD does not adequately describe all facets of the disease.

In 2014, KDIGO identified that the term MCKD and unregulated usage of replacement terms (familial juvenile hyperuricemic nephropathy, hereditary interstitial kidney disease, etc.) were especially misleading and had led to incorrect diagnoses, missed diagnoses, and overall decreased knowledge of the disease [40-41]. Situations such as patients receiving vague diagnoses regarding their condition, physicians having been taught incorrect information, and thus, having poor overall disease understanding are all due to poor disease characterization. Therefore, with the goals of elucidating the predominant four genotypes that result in MCKD, separating the clinical and diagnostic features, and creating a standardization of indications, interpretations, and technical aspects, the consensus proposed the term ADTKD as the replacement. One caveat, however, was that the terminology reflected the clinical presentation as a disease instead of its recognition as a syndrome. In terms of pure technicality, a disease signifies a definite cause with clear symptoms and treatment, while a syndrome is a collection of symptoms that may have more ambiguity in cause, presentation, and treatment. Eckardt et al. argued in 2015 that, in addition to practical reasons for a grammatically singular terminology, ADTKD also shares similar features with autosomal polycystic kidney disease, a pathology that is also termed a disease despite multiple etiologies [42]. As the name ADTKD can be slightly convoluted, patient-centered language in clinics, such as mucin-1 kidney disease or uromodulin kidney disease (formerly called MCKD1), was suggested to be introduced in conjunction with ADTKD. This was in part guided by the overall goals of the 2019 KDIGO conference, in which the revision of kidney terminology was to focus on patient-centeredness and precision. Therefore, names such as ADTKD-UMOD, ADTKD-MUC1, ADTKD-REN, and ADTKD-HNF1B are likely to be used more frequently in the future.

Perspectives on implementation

The implementation of new nomenclature across various healthcare systems could present challenges that must be addressed. There is a large diversity in global systems with differences in the availability of resources, access to genetic testing, and educational infrastructure. International adoption of a new nomenclature would be difficult to facilitate. Mitigating this aspect would focus on the development of international guidelines that would detail protocols for diagnostic criteria, clinical features, and genetic testing. These guidelines would then be disseminated through professional societies and global health organizations. Patient-centered communication and targeted education for healthcare professionals would be at the forefront in implementing the proposed nomenclature through various workshops, webinars, patient information materials, and integration into Electronic Health Records.

Additionally, perspectives regarding the proposed nomenclature change need to be considered when highlighting the rationale underlying the proposed new nomenclature. For healthcare providers, the new nomenclature would warrant comprehensive training, medical records updates, and treatment protocols. The feedback from healthcare providers is necessary in finalizing any decisions to ensure practicality in clinical settings. Additionally, patients may benefit greatly from the nomenclature change with a potential reduced stigma and confusion of ADTKD compared to the previous term of “MCKD” due to clinical manifestation variability and potential misdiagnoses. Involving patients in the decision-making process with focus groups or surveys is important to ensure that any new nomenclature recognizes patients’ perspectives as well as appropriately reflects their experiences in the healthcare they receive. To close this loop in the healthcare system, policymakers’ perspectives should also be considered. In response, policymakers must be willing to coordinate with healthcare providers and patient groups to ensure proper transition and adoption of new terminology while also preparing for updated medical guidelines and related insurance plans’ adjustments. The inclusion of perspectives from healthcare providers, patients, and policymakers on the proposed nomenclature change will hopefully enhance clarity, practicality, and the overall patient-centered healthcare approach.

## Conclusions

ADTKD, a more specific term reflecting both the genetic basis and the related pathogenesis, is ultimately aimed at providing more precise knowledge and valid awareness of this kidney disease among patients, clinicians, and researchers. Due to the predominant clinical feature of tubular fibrosis rather than cysts in the kidney medulla, the MCKD term is to be removed from all terminology due to its misleading nature. It is believed that this name change will enhance future pathophysiological research and disease population observations through a more uniform terminology. In addition, with the new terminology, enhanced diagnostic accuracy, it can be speculated that the estimation of the actual prevalence of the disease will significantly improve. While the adoption of ADTKD is pushed, as more research and diagnostic criteria are established in the spirit of the 2019 KDIGO committee guidelines, the name will remain open to revision to reflect evolving contemporary knowledge more accurately.
